# Non-Participation in Breast Cancer Screening in Spain and Potential Application in the Present and Future: A Cross Sectional Study

**DOI:** 10.3390/cancers13174331

**Published:** 2021-08-27

**Authors:** Juan José Muñoz-Sanz, María Jiménez-Palomares, Elisa María Garrido-Ardila, Juan Rodríguez-Mansilla

**Affiliations:** 1 Merida University Center (Badajoz), Department of Nursing, Extremadura University, 06800 Badajoz, Spain; jjmunoz@unex.es; 2 ADOLOR Research Group, Department of Medical-Surgical Therapy, Faculty of Medicine and Health Sciences, Extremadura University, 06006 Badajoz, Spain; mariajp@unex.es (M.J.-P.); jrodman@unex.es (J.R.-M.)

**Keywords:** breast, cancer, screening, non-participation

## Abstract

**Simple Summary:**

Breast cancer screening programmes have the aim of reducing breast cancer mortality. This article is an observational, descriptive, cross-sectional and retrospective study of 2507 women who were invited to participate in the Breast Cancer Prevention Programme in Extremadura (Spain) and did not attend their appointment. We analysed the different reasons why women do not participate in the Breast Cancer Early Detection Programme in Extremadura (Spain) and discuss the results offering possible tools to improve the screening programs. Women who did not participate in the breast cancer screening programme in Extremadura had low educational levels and were older women.

**Abstract:**

Background: Currently, we are beginning to observe a stabilisation and even a decrease in breast cancer mortality in the world, which may be related, among other reasons, to breast cancer screening. Methods: The objective of this study was to analyse the different reasons why women do not participate in the Breast Cancer Early Detection Programme in Extremadura (Spain) and to discuss the results, offering possible tools to improve the screening programs. This is an observational, descriptive, cross-sectional and retrospective study. A questionnaire with 14 questions was carried out by telephone or mail. Results: A total of 3970 questionnaires were collected. However, only 2507 were valid. A total of 70.36% of young and educated women underwent mammographic controls. The type of women who did not attend the screening programme appointment corresponded to a woman of approximately 60 years of age, with no formal studies, married, with children, who does not work outside their home and who lived in the health area of Badajoz. Among the main reasons for not going to the appointment, 53.9% of the women surveyed indicated that they had check-ups with their gynaecologist, and this specialist referred them for a mammograph. These women were younger and have a higher level of education. Women with a lower educational level and older women did not have any mammography done and did not undergo screening. They indicated that they did not go to the appointment because they were afraid of having a mammography (44%) or because they did not receive the appointment in time (31.6%). A total of 26.9% of the women who did not attend the appointment for other reasons stated that they had problems in attending because they had a physical limitation (dependency). Conclusions: Women who did not participate in the breast cancer screening programme in Extremadura had low educational levels and were older women. Specifically, fear of having a mammogram was the main argument raised by these women. In addition, a small group stated that they did not consider mammography to be useful. At present and in the future, good quality screening programs must be carried out to contribute to the reduction in breast cancer mortality. Furthermore, enhancing the participation of women is essential to increase the attendance rate and, therefore, the success of the screening programmes.

## 1. Introduction

Worldwide, breast cancer is the most common cancer in women [1]. In Spain, it represents between 20% and 25% of all tumours. Its mortality rate ranges from 17.6 to 60.2 per 100,000 women [2].

The course of the disease is well defined. The period of time between the appearance of the first lesions in the breast and the systemic spread of the disease takes an average of 10 to 15 years, which may vary according to the type of tumour and the age of presentation. There is a detectable preclinical phase, which lasts between 1 and 3 years [3,4], and survival varies greatly according to the stage of the cancer. It is known that the survival of a patient with breast cancer without lymph node involvement reaches 85% at 5 years. However, when the nodes are invaded, the survival drops to 53% or less. These figures justify the importance of the early diagnosis of the tumour [1,4,5,6].

Currently, a decrease in breast cancer mortality has been observed in the world. For some authors, this change is related to breast cancer screening, among other reasons [6,7]. However, in recent years the controversy over the effectiveness of breast cancer screening programmes (BCSP) has been the subject of major studies, meta-analyses and conferences [8]. Numerous clinical trials have been conducted to evaluate the efficacy of the early detection of breast cancer by mammography. Their results have shown that the screening for this disease and the early treatment can reduce mortality [9] and that accurate diagnosis can even be made in women in their 40s [7]. Therefore, at the present time, for prevention reasons, it is essential to know in-depth the levels of non-participation of women invited to undergo a mammographic study in breast cancer screening programmes and its characteristics. In this respect, studies have started to be conducted in order to assess the reasons for the non-participation of these patients in breast cancer screening programmes, although according to experts, they are scarce and insufficient [10]. Particularly in Extremadura, there are no data available on non-participation in the breast cancer screening programmes as there are no studies in the literature that evaluate this aspect.

The available scientific literature suggests some of the reasons that could explain the non-participation of women in the screening programmes. These reasons include the relationship between the cultural and socioeconomic level of women with the attendance to the screening appointments [11,12,13,14,15] or with the fact that patients with breast cancer, who have a high risk of developing a new primary cancer, showed low participation because they were already receiving treatment for the disease [14,16].

The European Union, in its “Europe Against Cancer” programme, recommends the implementation of these population-based screening programmes [17]. In the last ten years, the mortality rate of breast cancer has fallen by 12% in the European Union, and so has the number of women diagnosed each year [17].

In general, cancer mortality in Spain and in Europe has experienced a substantial decline in recent decades. The scientific evidence indicates that this tendency reflects the improvements in the survival of patients with tumours due to preventive activities, early diagnosis campaigns, therapeutic advances, and, in men, a decrease in the prevalence of smoking [18].

In Spain, specifically in the region of Extremadura, a breast cancer early detection programme has been carried out since 1998. This programme has the aim of reducing breast cancer mortality by 30% in 10 years, from the beginning of the programme, and improving the survival of affected women by increasing the rate of diagnosis in early stages from 15% to 30% [3]. Determining which are the main causes why the screening programme has not achieved its main objectives (high participation and reduction of cancer mortality) is fundamental to evaluate the effectiveness of these programmes.

In relation to the population screening in Spain, the guidelines are set independently by each of the local governments in the 17 regions of the country. There are some homogeneous criteria supported by the scientific evidence, but each regional government choose the citation system, the age of the target population and the system for analysing the results.

In the case of Extremadura, the target population was initially established in women between 50 and 64 years of age. Women between 40 and 49 years of age with a first-degree family history of breast cancer can also be included in the programme. The screening is monitored by the Women’s Programs Unit of the General Directorate of Public Health of the Extremadura Health Service. A female physician is in charge of the service and is responsible for organising the routes to be taken by the mobile vehicles and acts as the coordinator of the different radiological units for reading the results. All the results are communicated to the general practitioners assigned to the patients so that they can inform them [3].

When the regional government of Extremadura received the complete competencies in health matters from the State (1 February 2002), the authors of this study suggested to the competent authorities the need to evaluate the functioning of the programmes that were going to be managed directly by the regional government itself. In particular, the participation rate of the breast cancer screening was considered to be well below what is desirable and expected in the programme’s objectives [3].

Based on all this, the objective of this study was to analyse the different reasons why women do not participate in the Breast Cancer Early Detection Programme in Extremadura (Spain) and to discuss the results at a practical level, offering possible tools to improve participation in future screening programs.

## 2. Materials and Methods

### 2.1. Study Design

This is an observational, cross-sectional, descriptive and retrospective analytical study. The study population was women who did not participate in the Early Detection of Breast Cancer programme in the autonomous community of Extremadura (Spain) during 2002. The study was registered in the Territorial Registry of Intellectual Property, Regional Ministry of Culture, Extremadura, Spain (Registration No. 14/2004/96). All the ethical considerations and requirements mentioned in the Helsinki declaration [19] and the Data Protection Law [20] were met. The subjects included in the study signed the informed consent form to participate in the research.

### 2.2. Procedure

The study was carried out in the following phases:

1. Extraction of the data corresponding to the population under study and selection of the sample. The records corresponding to the women who did not respond to the invitation to participate in the Early Detection of Breast Cancer programme in the period from 1 January to 31 December 2002 in the region of Extremadura (Spain) were extracted.

A total of 17,127 records that met this condition were found. The records contained the following data: name and surname, address, telephone number, city/town of residence and date of birth. These data were exported to an Access^®^ database for processing and cleaning.

A random sampling with proportional sampling allocation was carried out. We proceeded to obtain the number of cases in the total population clustered by locality of residence, obtaining the representativeness values for each one of them. Based on these values, we estimated the realisation of 2500 surveys. Once the final number of surveys to be completed and the total population of each of the localities was known, we applied the proportional allocation equation, which allows us to know the number of surveys to be carried out in each of them:

N/n = N1/n1

N = total population; n = sample (total number of surveys); N1 = population of the locality; n1 = sample of the locality.

We estimated that the results obtained were achievable, and we established the objective of obtaining 2507 sample units to carry out the study.

One of the important problems of surveys or questionnaires is the low response rate obtained. This could be due to loss of information or to the lack of collaboration, which can cause the sample used to collect information to end up not being representative of the population [21]. For this reason, and in order to guarantee the representativeness of the study population, we considered it necessary to set the number of complete surveys to be carried out at 2507. For the selection of the sampling units, we carried out a random sampling in each of the localities by randomly choosing the first sample element, applying an elevation index. This method ensures that elements of all the classes that make up the study population appear so that it can generate more representative samples than simple random sampling.

In cases where the surveys were unsuccessful or incomplete, a new case was substituted in the same locality, following the same procedure.

2. Survey design and validation. We conducted a descriptive survey with a structured questionnaire so that all participants were asked the same questions in the same way and in the same order, thus guaranteeing the homogeneity of the responses and facilitating their subsequent computer processing [21]. The questionnaire can be found in the supplemental material.

The first step of the design was consulting the existing literature and studies with similar characteristics [11,22,23,24] in order to consult the survey models used and their possible translation to our study and the adaptation to the population of the region of Extremadura. The appropriate language was used in the wording of the questions.

The questionnaire contained 14 questions, which were clustered into: awareness of the programme, reasons for non-participation, attitudes or beliefs about mammography and characteristics of the respondents (socioeconomic, cultural and age variables).

Once the survey was designed, we proceeded to print 50 surveys for validation. This allowed us to detect possible errors when obtaining the information or problems in the interpretation of the questions, which could make the final result of the survey invalid. This process was carried out on 50 women from the total sample population who, after this test, were excluded from the final sample.

The data collection system was performed by telephone interview or postal mail, which were conducted by two surveyors. They were external to the research, trained in the methodology and resolution of common problems in order to guarantee a homogeneous interpretation of the responses.

The only tool available to us for the selection of women is the municipal census—the list of woman residents in each town of Extremadura. The common field in all these lists is the postal address, so the most direct way to reach all of them was through a written letter. When the lists also provided the contact telephone numbers, the survey was expedited by a telephone call [25].

The surveys that were sent by mail were accompanied by a letter from the Regional Minister of Health and Consumer Affairs of the Regional Government of Extremadura, informing about the reasons for the survey, requesting participation (informed consent) and providing the means for the participants to send back the completed survey.

Two telephone lines were set up for conducting the surveys at two workstations equipped with desktop PCs connected to a network. A total of 950 surveys were sent by mail, and we established a waiting period for a response of 45 days. In addition, 1557 telephone surveys were conducted. All surveys, both by telephone and by mail, were carried out during 2003, from March to October.

Finally, the anonymous treatment of the data was carried out, eliminating the personal data of the respondents, once the completion of the surveys under study by the trained interviewers was concluded.

The authors of this study designed the database where the information from the surveys was transferred. For this purpose, the Microsoft Access^®^ 2016 computer application was used. A form was created for data transfer. It had restrictions by value mask and selection filters that prevented the recording of erroneous data or incorrect answers. Access to the database was restricted by passwords so that no one unrelated to the study could access the data.

### 2.3. Data Cleaning

The results of the questionnaires were cleaned and analysed by the principal investigators. Before starting to transfer the data into the statistical application as the first step of the analysis, we eliminated the personal data contained in the initial table, retaining only the city or town of residence and date of birth because of their interest in the study. Finally, we exported the 2507 valid and complete records that were subjected to analysis and assessment.

### 2.4. Statistical Analysis

The statistical analysis was started by performing a descriptive analysis of each of the questions. The appropriate methods to carry out the proposed objectives are framed in the Categorical Data Analysis (Contingency Tables).

To study the degree of the relationship between two variables, we used measures of association that attempt to quantify the degree of relationship by eliminating the effect of the sample size.

Two measures based on the chi-square test (Phi and Cramer’s V) were used. They provide a measure of how much the observations vary from the mean values expressed in the same units as the data.

The statistical residual of a cell was calculated as the observed value minus the predicted value, although there are different variations. The most used residuals were the so-called Haberman corrected typed residuals, calculated as the raw residual divided by an estimate of its standard error.

In a second phase, we aimed to locate, geographically or based on other aspects, those groups of women who might not be undergoing any type of radiological control. Therefore, we used descriptive methods on selections of cases based on the survey itself, which allowed us to approximate the profile of the groups of women to be determined, including frequency tables.

The Microsoft Access version 2016 was used for the data transfer and database management. All the analysis was carried out with the SPSS v.22 statistical package in Spanish.

## 3. Results

Out of a total of 3970 surveys collected, 2507 were valid. The exclusion criteria are shown in Table 1.

It should be highlighted that 52.2% of the invalid surveys corresponded to women who refused to answer the questions. The rest of the causes, which represent 47.8% of the data, are issues related to failures of the information systems. Among these, and eliminating cases purely related to postal data, it is worth mentioning that 30 women had already died, and 12 men were invited to participate in the programme.

In the study, a random sampling with proportional allocation to the universe population was performed. This provided a fairly representative sample of the overall population. We assume that our universe population (more than 17,000 women) is a demographically representative sample of the general population (corresponding to more than 40% of the total number of women targeted by the programme) [21].

The results of the first descriptive study (Table 2) showed that the type of woman who did not attend the Breast Cancer Early Detection Programme appointment corresponded to a woman of about 60 years of age, with no formal education, married, with children, who do not work outside the home and who lives in the health area of Badajoz.

Among the main reasons for not attending the appointment (Table 3), 53.9% of the women surveyed indicated that they had check-ups with their gynaecologist, and it was this specialist who requested the mammogram. These women were younger and had higher education. Seventeen percent said they did not receive the appointment in time, and 13.6% were afraid of having a mammogram.

Focusing on the 743 women in the sample who did not have a mammogram and were not screened (Table 4), we found that the main reason given by this group for not attending the appointment was being afraid of having a mammogram (44%), followed by not receiving the appointment in time (31.6%) and having problems with transport (15.2%).

Analysing the women who reported being afraid of the mammogram, we found that the characteristics of this sample corresponded to women with lower educational levels and older age (Table 5).

Our results (Table 3) also showed that 60% of the study population was undergoing mammography controls. We wanted to check the profile of this group of women and found that they corresponded to younger women with formal education (Table 6).

On the other hand, among the women who indicated that they had a different reason for not attending the appointment, it should be noted that 26.9% of the cases corresponded to women who stated that they had problems in attending because they had a physical limitation (they were on a situation of dependency) (Table 7).

## 4. Discussion

This study provides an important insight into the reasons why women do not participate in the Breast Cancer Early Detection Programme.

The interest in achieving a high participation rate in breast cancer screening programmes is persistent [26]. Women’s participation in these programs depends mainly on the women’s attitudes and the previous knowledge about breast cancer screening [27], the role of health professionals in raising awareness [28] and reinforcing the indication to participate, the different recruitment systems (postal and telephone communication systems to give appointments) or the location of the mammography units [29].

In our study, and after obtaining a participation rate in the Early Detection of Breast Cancer Programme in Extremadura of 63.84% in the first round of screening (according to results presented by the Ministry of Health and Consumption of Extremadura), we considered the need to carry out a detailed survey that allowed the analysis of the reasons why some women in our region do not go for mammograms, to study the characteristics of this group of women and, therefore, to improve participation in future interventions.

It was necessary to complete 3970 questionnaires to obtain the 2507 surveys that formed the sample. A total of 36.9% of the women did not complete the survey, of which a high percentage was due to failures in the database. Out of the total number of failed surveys, 12.7% corresponded to women who had undergone mammograms, which can be interpreted as duplicities in the database. In this respect, as in other studies [11,30,31], frequent errors in the population database have made it difficult to locate many women included in the target population

In our database, there were a large number of duplications, which obviously lowered the participation rate. In addition to the duplications, there was also a high percentage of women who were not included in the database. In the study, 44.6% had not received the appointment in time, and, among these, 97.2% stated that they had not received any letter.

Therefore, in order to achieve and maintain high participation rates, individual citation systems based on valid and reliable population-based records that are continuously evaluated and updated must be used. Without a well-defined and identified population, it is not possible to assess the quality of a screening programme and its effectiveness [32].

In addition, to decreasing the participation rate, we believe that a woman receiving multiple appointments for mammography screenings may detract from the credibility of future screening programmes.

The main reason why women did not go for mammography screening was that they followed a regular check-up with the specialist who requested the test. This data coincides with other studies where women under treatment or medical supervision did not attend screening programmes [16]. In our study, 55.3% of the women (1387) had a mammogram done the previous year, so they were no longer eligible for the screening test and, therefore, did not attend the appointment. If we were to add to our participation rate the women who did not participate, for this reason, it would clearly exceed the 70% needed to ultimately reduce breast cancer mortality. It would be interesting to determine what percentage of these women actually needed screening since, among the 1714 women who underwent regular mammography, only 30.1% (516 women) reported having breast problems.

The fact that these women underwent periodic check-ups by a specialist and, in addition, that they did so in the hospital only increases the waiting lists and wastes the resources available to the Breast Cancer Early Detection Programme for this purpose.

The activities and procedures that would facilitate women’s acceptance of the invitation to participate in the screening include training, information and awareness-raising activities, the participation and training of all health professionals, the cooperation of citizen associations in the dissemination of the programme and the use of the media [30].

Primary care teams [33,34] have a privileged position to identify and contact the target population of women who can benefit from breast cancer screening. However, it seems to us that it would be essential to also involve the specialised care teams. The profile of women who did not attend because they were already assessed and followed up in other health centres seems to be related to a certain attitude and information regarding the use of health services and clearly coincides with those women with middle or higher education [11,30,35] and women who are younger [11,30,35,36,37].

In our study, as in that of Segura et al. [30], although participation increased with educational level, it was slightly lower at higher educational levels. We observed that women with higher educational levels are those who perform more activities outside the home (work outside home) and also coincides with the population group that most frequently attend private consultations.

Health services are also less demanded by single women with respect to other marital statuses. Among the single women in our study, 44.7% reported not having any type of mammography check-up, mainly due to the fear of results, difficulties travelling to the mammography unit or not receiving an appointment in time.

Our results indicate that women’s fear of the results of the mammogram seems to be the main reason why they did not follow a periodic control, the second reason was not receiving the appointment, and the third reason was the difficulty in accessing the test. 

The contribution of the primary care physician is to provide information, advice and reassurance [38], in addition to recommending screening. Studies conducted in healthcare staff have found that knowledge, attitudes and practices related to breast cancer screening were lower than expected, so it is recommended to increase information in that aspect [28].

It is very important for women to be aware of the benefits of undergoing mammography in the early stages in the event that they might be affected by breast cancer [7]. This lack of information is evident when they were asked why they are afraid of the mammogram; most of them answer that they did not care if they knew the result of malignancy earlier or later.

Currently, the appointment system used by the Breast Cancer Early Detection Programme in Extremadura consists of a previous invitation, by postal letter, which is accompanied by a leaflet with general information about the programme. It would be interesting if, in the invitation to participate, the woman was advised to consult her physician for any doubts or ask for advice if she is not familiar with the programme or has questions regarding participation or results.

Only 7.5% of the women (187) reported having problems travelling to the mammography unit, although this is an important reason for women who did not undergo regular check-ups. Those who lived in towns with no hospital, nor a mobile unit, were the most affected, although the Breast Cancer Early Detection Programme provides a subsidy to the municipalities of these towns to facilitate collective transportation for these women. In this aspect, we coincide with other available research that also emphasises that a limitation to participate in screening programmes is accessibility [10].

Regarding the existence of other reasons for not attending, an important group (43 women) stated that they had physical limitations or impairments. This group mainly lived in localities with access to a hospital or mobile unit, which makes us consider the need to facilitate access to people in a situation of dependence, when necessary.

A large number of factors may determine the final decision about participation: the existence or not of an established screening programme, the previous exposure to mammography screening, the cost of mammography, the involvement of health professionals, the availability of sufficient resources and logistical support, the dissemination of information campaigns, the efforts of social entities and associations and the influence of age, social and educational status, among other nonspecific elements [39].

A population screening programme must meet the criteria of acceptable cost-effectiveness and, fundamentally, it must be guaranteed to be carried out over the years and at the planned time intervals [32].

The stability and potential prestige of community-based breast cancer screening programs are also elements that can contribute to the dissemination of information and encouraging participation. [39].

The effectiveness and the benefit of the screening programmes is really measured by the decrease in mortality. For this, very good quality breast cancer early detection programmes that achieve the expected reduction in breast cancer mortality must be carried out and should be developed over at least a ten-year period [40].

If the participation rate remains within the objectives, it is foreseeable that the effectiveness of the programme will be as expected, which is why it is essential to intensify quality control mechanisms in the screening programs [40].

It would also be necessary for future research to carry out a cross-sectional study at the national level in order to know all the data in the country concerning the non-participation of women in screening programmes. However, the absence of published data in each of the regional governments and the lack of homogeneity of criteria among the different screening programmes (different age limits in the target population, differences in citation and recruitment systems, differences in reading systems or validation of results) could make this task difficult.

### Limitations of the Study

We consider that the main limitation of the study is the lack of nationwide data available in relation to the non-participation in breast cancer screening. Another limitation that could be considered is the period of study as we only conducted the study with women who have not participated in the programme during a one-year period. However, it would have been interesting to compare some variables (age, marital status, children, activity outside the home, fear of undergoing mammography, accessibility to the mobile unit, etc.) with the population group that did attend the screening test during the same period.

Although validation of the scale with the study population was performed, a quantitative treatment of the responses (using scales or similar) would have greatly facilitated the process of analysing the results. One of the major difficulties encountered in this study, from the methodological point of view, was the analysis of categorical variables that required a bibliographic and statistical effort much greater than expected.

Another limitation was the fact that the rate of failed surveys was not studied independently according to the system of contact with the woman surveyed. This was because the data in the information systems consulted was not up to date, including both the postal address and the contact telephone number [32].

## 5. Conclusions

The majority of women in the region of Extremadura reported having some type of mammographic control, either by check-ups with their gynaecologist or by a mammogram in the last year. This behaviour is more accentuated in younger women with a higher level of education.

The main problems that cause the non-attendance at the Breast Cancer Early Detection programme in Extremadura were the lack of coordination between healthcare levels (Primary Health Care/Hospital Care), low health education, failures in the information systems and accessibility problems.

Women who did not report undergoing any type of mammographic screening were older women with lower educational levels. In particular, the fear of having a mammography was the main argument provided by these women. In addition, a small group also stated that they did not consider mammography to be useful. The second reason for not attending the screening was not receiving the appointment in time. Problems in travelling to the mammography unit showed a high prevalence, being the first reason for non-participation in the programme for some of the participants.

Good quality screening programmes must be carried out in order to achieve the expected reduction in breast cancer mortality, for which the participation rate must be increased. In order to achieve this, information and participation actions should be promoted to publicise the programmes and the benefits of the early detection of this pathology.

## Figures and Tables

**Table 1:** Results of the invalid surveys.

Result of the Survey	Frequency	Percentage
Incomplete (did not answer all questions)	90	6.1%
Failed (survey not done)	1373	93.9%
Cause of the Invalid Survey		
She had the mamography done	174	12.7%
Refuses to answer	716	52.2%
Deceased	30	2.2%
Untraceable	23	1.7%
Incorrect number	228	16.6%
The person contacted was a man	12	0.9%
Returned letter	49	3.6%
Absent	141	10.3%
Total	1373	

**Table 2:** Socio-demographic data of the women who did not attend the programme.

Variables	Frequency	Percentage
Level of education		
No formal studies	1354	54.0%
Primary school	853	34.0%
Secondary school	94	3.7%
High school	92	3.7%
University studies	114	4.5%
Age group		
50 to 54 years old	415	16.6%
55 to 59 years old	823	32.8%
60 to 64 years old	831	33.1%
Over 65 years old	438	17.5%
Marital status		
Single	168	6.7%
Married	2087	83.2%
Widowed	207	8.3%
Separated/Divorced	44	1.8%
Other	1	0%
Do you have children?		
No	236	9.4%
Yes	2271	90.6%
Do you work outside home?		
No	1954	77.9%
Yes	553	22.1%
Health zone		
Badajoz	927	37.0%
Mérida	328	13.1%
Don Benito-Villanueva	228	9.1%
Llerena-Zafra	204	8.1%
Cáceres	490	19.5%
Coria	93	3.7%
Plasencia	132	5.3%
Navalmoral de la Mata	105	4.2%
Province within the region of Extremadura		
Badajoz	1687	67.3%
Cáceres	820	32.7%

**Table 3:** Reasons for not attending the appointment.

Cause	Frequency	Percentage	Asymptotic Sig. (Bilateral)
Mammograph the previous year	152	6.1%	0.000
Gynecologist check-up and mammogram	1352	53.9%	0.000
Did not receive the appointment in time	425	17.0%	0.000
She thinks the mammogram is painful	2	0.1%	0.000
She is afraid of the test	341	13.6%	0.000
She thinks that it is not useful	43	1.7%	0.000
She had no means of transport	149	5.9%	0.000
She forgot that she had an appointment	43	1.7%	0.000
	Value	df	Asymptotic Sig.
Pearson’s Chi-square	106,273	21	0.000
Likelihood ratio	108,137	21	0.000
Linear by linear association	40,436	1	0.000

Note: Sig: Significance; df: Degree of freedom.

**Table 4:** Reasons for not attending the appointment of women who do not follow the screening.

Cause	Frequency	Percentage
Did not receive the appointment in time	235	31.6%
She thinks the mammogram is painful	2	0.3%
She is afraid of the test	327	44.0%
She thinks that it is not useful	43	5.8%
She had no means of transport	113	15.2%
She forgot that she had an appointment	23	3.1%
TOTAL	743	

**Table 5:** Socio-demographic data of women that were afraid of undertaking a mammogram.

Are You Afraid of the Mammography?	Level of Education					Total
	No Formal Studies	Primary School	Secondary School	High School	University Studies	
Yes	483	210	17	12	21	743
Are you afraid of the mammography?	Age group					Total
	50 to 54 years old		55 to 59 years old	60 to 64 years old	Over 65 years old	
Yes	91		202	275	175	743

**Table 6:** Socio-demographic data of women that underwent screening.

Did You Have a Mammogram or a Check up the Previous Year?	Level of Education					Total
	No Formal Studies	Primary School	Secondary School	High School	University Studies	
Yes	714	560	71	71	88	1504
Did you have a mammogram or a check up the previous year?	Age group					Total
	50 to 54 years old	55 to 59 years old		60 to 64 years old	Over 65 years old	
Yes	299	550		454	201	1504

**Table 7:** Other reasons for not attending the appointment.

Cause	Frequency	Percentage
She forgot the appointment	10	7.7%
She was ashamed	3	2.3%
Reluctant or not interested	36	27.7%
An unforeseen event came up	18	13.8%
Due to physical limitation (disability)	35	26.9%
Other reasons	28	21.5%
TOTAL	130	

## Data Availability

The data underlying this article cannot be shared publicly to maintain the privacy of individuals that participated in the study. The data will be shared upon reasonable request to the corresponding author.

## Supplementary Materials

The following are available online at https://www.mdpi.com/article/10.3390/cancers13174331/s1, S1: Mammography survey—Questionnaire used in the study (translation into English and original Spanish version).
