# Supplementary material for: Non-Participation in Breast Cancer Screening in Spain and Potential Application in the Present and Future: A Cross Sectional Study
Source: Cancers (Basel). 2021 Aug 27;13(17):4331. doi: 10.3390/cancers13174331 (PMC8430829; doi:10.3390/cancers13174331)
Supplement: Supplementary file 1 [file cancers-13-04331-s001.zip › cancers-1333697-supplementary.pdf]

SUPPLEMENTAL MATERIAL

Mammography survey - Questionnaire used in the study
(translation into English and original Spanish version)

PERSONAL INFORMATION

1. Education:
   a. No studies
   b. Primary school
   c. Secondary school
   d. Baccalaureate/vocational training
   e. University studies
2. Marital Status:
   a. Single
   b. Married
   c. Widowed
   d. Separated/Divorced
   e. Other____________________
3. Do you have any children?
   a. Yes
   b. No
4. Do you work outside the home?
   a. Yes
   b. No
5. Does the mobile mammography unit come to your town?
   a. Yes
   b. No
   c. Don't know

MAMMOGRAPHY SURVEY:

6. What do you consider to be the most important reason why you did not go for a mammography study? (please indicate only one reason):
   a. You had a mammography in the previous year
   b. You have regular check-ups with your gynaecologist, who referred you to undertake a mammography.
   c. You did not receive the appointment on time
   d. No one has told you what the test consists of and you think it may be painful or harmful to your health.
   e. You are afraid
   f. You think that having a mammography is useless.
   g. It was totally impossible for you to travel to the location where the Mobile Unit was located.
   h. You forgot that you had to go

7. Did you have a mammography last year?
    a. Yes
    b. No
    - If you answered yes, where was it done?
        i. At the hospital
        ii. In a private practice
        iii. In the Breast Cancer programme
8. Do you have regular mammographys?
    a. Yes
    b. No
    - If yes, where is mammography usually performed?
        i. In the public health system
        ii. In the private sector
    - Did you have mammographys because you have breast problems?
        iii. Yes
        iv. No
9. Did you receive your appointment on time for your mammography?
    a. Yes
    b. No
    - If you answered that you did not receive the appointment on time, can you please indicate the cause?
        i. Absent from home for some time
        ii. Changed address
        iii. The appointment arrived but late
        iv. Did not receive any letter
10. Are you afraid of having a mammography?
    a. Yes
    b. No
    - If you answered yes, can you explain why you are afraid?
        i. No one has informed you what it consists of
        ii. You think that it may hurt you or that it may harm your health
        iii. You are afraid of the results
        iv. Other causes: _____________
11. Do you think having a mammography is helpful?
    a. Yes
    b. No
    - If you answered that you do not think it is useful, could you say why?
        i. Because if the result is bad, you don´t care if you have the mammography done before or after.
        ii. Because on one occasion you were told that the mammography showed 'doubtful results' and finally, there was nothing wrong.
        iii. Because an acquaintance of you was told that the mammography was normal and it turned out that she had a tumor.
        iv. You do not know

12. Did you have any problems travelling to the location where the Mobile Unit was located?
    a. Yes
    b. No
    - If you answered yes, what was the main reason that prevented you from travelling?
        i. Because you had no car and no one who could give you a ride.
        ii. Because you were not willing to travel to get a mammography
        iii. Because you could not leave your job
        iv. Other Reasons: ____________
13. Where do you prefer to have your mammography done?
    a. At the hospital
    b. At the mobile unit
    c. You don't mind
14. Is there any other reason or impediment why you did not go for a mammography?
    a. Yes
    b. No
    - If you answered that there was some other reason why you did not attend, could you please state what it was?:
        xxiv. You forgot the day you were supposed to go
        xxv. You are embarrassed
        xxvi. Out of reluctance, disinterest, don´t feeling like it.
        xxvii. An unforeseen event came up
        xxviii. Due to physical limitation (disability)
        xxix. Other causes: ______________

ENCUESTA SOBRE MAMOGRAFÍAS

FECHA: .... / .... /2003	ENCUESTA PCM 00000

DATOS IDENTIFICATIVOS

Nombre y Apellidos: ....

Dirección: ....

C.P.: .... Localidad: .... Provincia: ☐ BA ☐ CC

Teléfono: ....

RESULTADO DE LA ENCUESTA

☐ Completa

☐ Incompleta (se negó a contestar determinadas preguntas)

☐ Fallida → ¿Causa?:
- ☐ No localizada
- ☐ Ausente
- ☐ Se niega
- ☐ Fallecimiento
- ☐ Es un hombre

OBSERVACIONES:

....

....

....

....

....

....

....

....

....

PROGRAMA DE DETECCIÓN PRECOZ DEL CÁNCER DE MAMA

Consejería de
Sanidad y Consumo

Dirección General de
Salud Pública

JUNTA DE EXTREMADURA

PCM 00000

CUESTIONARIO

DATOS PERSONALES

1. Estudios terminados:
   - ☐ 1.1. Sin estudios
   - ☐ 1.2. Primarios (EGB primera etapa)
   - ☐ 1.3. Secundarios (EGB segunda etapa, bachillerato elemental)
   - ☐ 1.4. Bachillerato / FP
   - ☐ 1.5. Universitarios
2. Estado civil:
   - ☐ 2.1. Soltera
   - ☐ 2.2. Casada
   - ☐ 2.3. Viuda
   - ☐ 2.4. Separada / Divorciada
   - ☐ 2.5. Otros (especificar): ....
3. ¿Tiene hijos?:
   ☐ Sí ☐ No
4. ¿Trabaja fuera de casa?:
   ☐ Sí ☐ No
5. ¿Va a la Unidad Móvil de Mamografía de su localidad?:
   ☐ Sí ☐ No ☐ No sabe

ENCUESTA SOBRE MAMOGRAFÍAS

0. Señale cuál es la causa que considera **más importante** por la que no acudió a realizarse la **mamografía** (señalar sólo una causa):
   - ☐ Tenía realizada una mamografía en el año anterior
   - ☐ Hace revisiones de forma habitual con su ginecólogo y éste le solicita las mamografías
   - ☐ No recibió la cita a tiempo
   - ☐ Nadie le ha informado en qué consiste la prueba y piensa que puede resultar dolorosa o que perjudique su salud
   - ☐ Tiene miedo
   - ☐ Piensa que hacerse una mamografía no sirve para nada
   - ☐ Le fue totalmente imposible desplazarse a la localidad donde estaba la Unidad Móvil
   - ☐ Se le olvidó el día que tenía que ir
1. ¿Tenía realizada una **mamografía** el año anterior?:
   ☐ Sí ☐ No
   - *Si ha contestado que SÍ la tenía realizada del año anterior, ¿dónde la realizó?:*
     - ☐ En el hospital
     - ☐ En una consulta privada
     - ☐ En el Programa de Cáncer de Mama

Continúa al dorso

2. ¿Se realiza mamografías periódicamente?:

- [ ] Sí   - [ ] No

• *Si ha contestado que SÍ, ¿dónde se realiza la mamografía habitualmente?:*

- [ ] En la Sanidad Pública
- [ ] En la Privada

• *¿Se hizo las mamografías porque tiene problemas en las mamas?:*

- [ ] Sí   - [ ] No

3. ¿Recibió la cita a tiempo para realizarse la mamografía?:

- [ ] Sí   - [ ] No

• *Si ha contestado que NO recibió la cita a tiempo, ¿puede indicar la causa?:*

- [ ] Faltó de su domicilio algún tiempo
- [ ] Cambió de domicilio
- [ ] Llegó la citación, pero fuera de tiempo
- [ ] No ha recibido ninguna carta

4. ¿Tiene miedo de realizarse la mamografía?:

- [ ] Sí   - [ ] No

• *Si ha contestado que SÍ, ¿puede explicar por qué tiene miedo?:*

- [ ] Nadie le ha informado en qué consiste
- [ ] Piensa que le puede doler o que puede perjudicar su salud
- [ ] Teme al resultado
- [ ] Otras causas (indíquelas si lo cree oportuno): ______________________________

5. ¿Piensa que hacerse una mamografía es útil?:

- [ ] Sí   - [ ] No

• *Si ha contestado que NO cree que sea útil, ¿podría decir por qué?*

- [ ] Porque si el resultado es malo le va a dar igual hacérsela antes o después
- [ ] Porque en una ocasión le dijeron que se veía "algo dudoso" en la mamografía y luego no era nada
- [ ] Porque a una conocida suya le dijeron que la mamografía era normal y resultó tener un tumor
- [ ] No sabe

6. ¿Tuvo algún problema para desplazarse a la localidad donde estaba situada la Unidad Móvil?:

- [ ] Sí   - [ ] No

• *Si ha contestado que SÍ tuvo problemas, ¿cuál fue el motivo principal que le impidió desplazarse?:*

- [ ] Porque no tenía coche ni nadie que le pudiera llevar
- [ ] Porque no está dispuesta a desplazarse para hacerse una mamografía
- [ ] Porque no puede abandonar su puesto de trabajo
- [ ] Otras causas (indíquelas si lo cree oportuno): ______________________________

7. ¿Dónde prefiere realizarse la mamografía?:

- [ ] En el hospital
- [ ] En la Unidad Móvil
- [ ] Le da igual

8. ¿Existe alguna otra razón o impedimento por la que no asistiese a realizarse la mamografía?:

- [ ] Sí   - [ ] No

• *Si ha contestado que SÍ hubo alguna otra razón por la que no asistió, ¿podría decir cuál fue?:*

- [ ] Se le olvidó el día que tenía que ir
- [ ] Le da vergüenza
- [ ] Por desgana, desinterés, inapetencia,...
- [ ] Le surgió un imprevisto
- [ ] Por limitación física (minusvalía)
- [ ] Otras causas (indíquelas si lo cree oportuno): ______________________________
